# Raising Near-Infrared Photoluminescence Quantum Yield of Au_42_ Quantum Rod to 50% in Solutions and 75% in Films

**DOI:** 10.1021/jacs.4c11703

**Published:** 2024-10-03

**Authors:** Lianshun Luo, Zhongyu Liu, Abhrojyoti Mazumder, Rongchao Jin

**Affiliations:** Department of Chemistry, Carnegie Mellon University, Pittsburgh, Pennsylvania 15213, United States

## Abstract

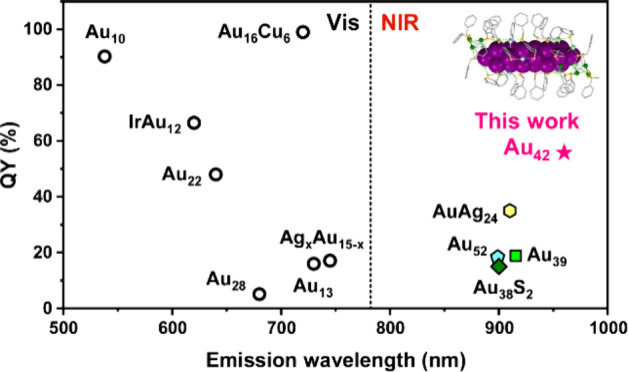

Highly emissive gold nanoclusters (NCs) in the near-infrared (NIR) region are of wide interest, but challenges arise from the excessive nonradiative dissipation. Here, we demonstrate an effective suppression of the motions of surface motifs on the Au_42_(PET)_32_ rod (PET = 2-phenylethanethiolate) by noncoordinative interactions with amide molecules and accordingly raise the NIR emission (875/1045 nm peaks) quantum yield (QY) from 18% to 50% in deaerated solution at room temperature, which is rare in Au NCs. Cryogenic photoluminescence measurements indicate that amide molecules effectively suppress the vibrations associated with the Au–S staple motifs on Au_42_ and also enhance the radiative relaxation, both of which lead to stronger emission. When Au_42_ NCs are embedded in a polystyrene film containing amide molecules, the PLQY is further boosted to 75%. This research not only produces a highly emissive material but also provides crucial insights for the rational design of NIR emitters and advances the potential of atomically precise Au NCs for diverse applications.

Luminophores emitting in the NIR region (800–1700 nm) window are increasingly valued across many fields,^1−5^ such as bioimaging and NIR optics.^6−8^ Thiolate-protected Au_*n*_(SR)_*m*_ NCs (SR = thiolate) have recently emerged as a promising class of NIR-emissive materials.^9−15^ These NCs feature a core–shell structure,^16−18^ in which the inner Au(0) core is enclosed by Au(I)–SR “staple motifs”. The tailorable size, structure, and composition of Au NCs allow them to exhibit emission peaks across the visible to NIR range.^19−22^ Moreover, their atomic precision aids in a deeper understanding of photophysical mechanisms,^23,24^ facilitating the design of highly luminescent materials. Currently, a few highly luminescent NCs in the visible range have been reported,^25−30^ but the NIR region is still difficult due to the energy gap law induced significant loss of excitation energy via nonradiative relaxation.^31,32^ The photoluminescence quantum yield (PLQY) of NIR-emissive Au NCs is often below 1%,^33,34^ except a few cases^15,21,35−40^ under ambient conditions.

Enhancing the PLQY can be accomplished by increasing the radiative decay rate (*k*_*r*_) and/or decreasing the nonradiative decay rate (*k*_*nr*_) according to the formula, . In the case of Au NCs, given their significantly higher *k*_*nr*_ (10^5^–10^7^ s^–1^) than the *k*_*r*_ (10^4^–10^5^ s^–1^), reducing the *k*_*nr*_ offers a greater opportunity for PLQY enhancement.^19,41,42^ The PL properties of Au NCs have been recognized to be intricately linked to the Au(I)–SR “staple motifs”, thus, restricting motions associated with these surface motifs is generally an effective strategy for achieving higher PLQY by suppressing the *k*_*nr*_.^5,43−45^

Here, we report a noncoordinating interaction strategy for the suppression of *k*_*nr*_ to enhance the NIR emission of rod-shaped Au_42_(PET)_32_ (PET = 2-phenylethanethiolate). Specifically, the nonradiative energy loss in Au_42_ is suppressed by the addition of amide-containing small molecules, thus improving the PLQY to 50% in solution at room temperature. Cryogenic PL analysis reveals that the vibrations associated with the Au–S staples on Au_42_ are suppressed by amide molecules. Moreover, when Au_42_ is embedded in a polymer film containing amide molecules, the PLQY is further boosted to 75% at room temperature.

The Au_42_ quantum rod was synthesized using a method of *N*-heterocyclic carbene (NHC)-mediated kinetic control reported by our group.^46^ The Au_42_ structure shows a rod-shaped, hexagonal close-packed Au_20_ kernel protected by two pairs of interlocked Au_4_(PET)_5_ motifs (marked in green and light green) on the two ends and six monomeric Au(PET)_2_ motifs (marked in blue) on the body (Figure 1A).^34,47^ The optical absorption spectrum of Au_42_ exhibits two major peaks at 375 and 806 nm (Figure 1B, green profile). Theoretical simulations identified that the 806 nm peak originates from the HOMO-to-LUMO transition and the transition dipole is strongly polarized along the longitudinal direction, while the 375 nm peak is not.^47^

**Figure 1:**
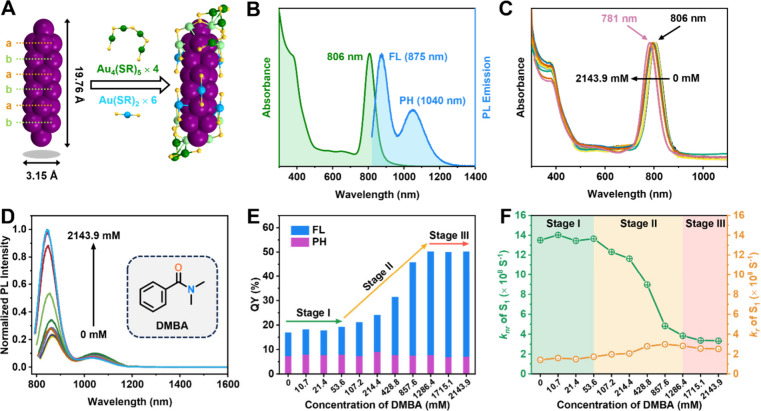
(A) Structure of Au_42_(PET)_32_. Color code: yellow = S, other colors = Au, carbon tails are omitted for clarity. (B) Optical absorption (green) and PL (blue) spectra of Au_42_ dissolved in C_2_Cl_4_. (C) Optical absorption spectra, (D) PL spectra, and (E) PLQY of Au_42_ dissolved in deaerated 2-MeTHF containing DMBA with different concentrations. (F) The *k*_*r*_ and *k*_*nr*_ of S_1_ state of Au_42_ in deaerated 2-MeTHF containing DMBA with different concentrations. For PL measurements: both slit widths 8 nm.

Upon excitation at 806 nm, Au_42_ exhibits fluorescence and phosphorescence dual emission at 875 nm (denoted FL) and 1040 nm (PH) (Figure 1B, blue profile), respectively, with a total PLQY of 18% (Figure S1); note that this value is higher than the 12% reported earlier^46^ due to the different excitation wavelengths (806 nm in this work versus 380 nm previously).

When Au_42_ (0.1 OD at 806 nm, absorption coefficient ε_806_ = 1.08 × 10^5^ M^–1^ cm^–1^,^48^ i.e., 9.26 × 10^–4^ mM) was mixed with nonluminescent *N,N*-dimethylbenzamide (DMBA, Figure S2), the Au_42_ absorption profile remains unchanged, but its NIR absorption peak blueshifts from 806 to 781 nm with increasing amide concentration from 0 to 2143.9 mM (Figure 1C and Figure S3), and the integrated PL intensity of Au_42_ increases significantly by ∼3-fold (Figure 1D and Table S1), reaching a total PLQY of 50.1% (Figure 1E and Table S1). Specifically, the PLQY initially remains unchanged with the concentration up to 53.6 mM (Stage I). It then exhibits a gradual rise, reaching 50.1% at the DMBA concentration of 1286.4 mM (Stage II), and maintains this intensity as the concentration is further increased (Stage III). When Au_42_ was precipitated out of the solution to remove amides and redissolved in C_2_Cl_4_, the PLQY of Au_42_ recovers to the initial 18%, indicating noncoordinative interactions between Au_42_ and DMBA.

The dual PL bands are deconvoluted to analyze the respective variation of FL and PH (Figures S4 and S5 and Table S1). It is evident that the FL shows a dependence on the concentration of DMBA, but the PH remains constant. Generally, the FL enhancement can be accomplished either by increasing the *k*_*r*_ and/or reducing the *k*_*nr*_. Here, our results reveal a significant reduction in the *k*_*nr*_ for the FL of Au_42_ upon the addition of DMBA, plummeting from 13.51 × 10^8^ s^–1^ to 3.33 × 10^8^ s^–1^, together with a moderate increase in *k*_*r*_ from 1.42 × 10^8^ s^–1^ to 2.51 × 10^8^ s^–1^ (Figure 1F and Table S1).

We further conducted cryogenic PL measurements from room temperature to 80 K (Figure 2A and B). For the Au_42_/DMBA system, we selected a DMBA concentration of 857.6 mM to ensure a significant PL enhancement but preventing the precipitation of DMBA at low temperatures. Given the fact that Au NCs exhibit stronger absorption at low temperatures, we also performed temperature-dependent absorption (Figure S6) to correct PLQY at low temperatures. The cryogenic PL for Au_42_ and Au_42_/DMBA in 2-methyltetrahydrofuran (2-MeTHF) are shown in Figure 2A-B. The PLQY of Au_42_ (without DMBA) increases from 16.8% to 45.6% as the temperature is lowered from 298 to 80 K; note: 16.8% in 2-MeTHF (“glass” forming solvent) slightly differs from 18% in C_2_Cl_4_. For the Au_42_/DMBA, the PLQY rises from 45.7% to 89.1% in the same temperature range. The detailed results of peak deconvolution are provided in Tables S2 and S3. Both FL and PH intensities for the two systems increase as the temperature decreases, in contrast to the sole FL enhancement by amide. The FL for the Au_42_/DMBA system is consistently higher than that of Au_42_ (Figure 2C). Conversely, the PH emission remains nearly identical for the two systems at each temperature, though the PH increases at lower temperatures (Figure S7). The PL excitation spectra for Au_42_ and Au_42_/DMBA were also compared (Figures S8 and S9). The PL excitation at 80 K shows a blue shift compared to that at 298 K, consistent with the cryogenic absorption (Figure S6A).

**Figure 2:**
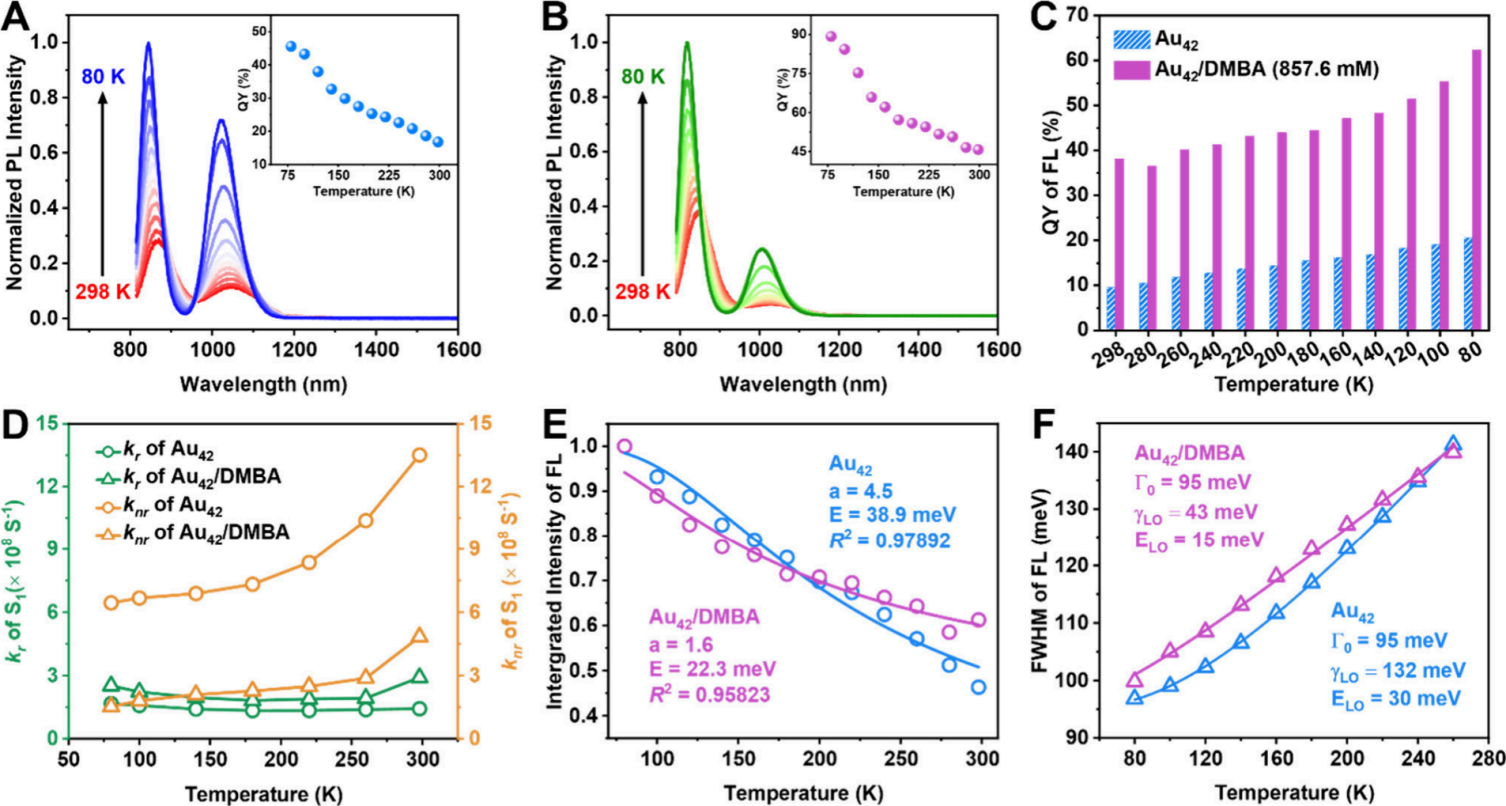
Temperature-dependent PL spectra of (A) Au_42_ and (B) Au_42_/DMBA (857.6 mM) in 2-MeTHF under a He atmosphere. Inset: the variation of total PLQY as the temperature decreases from 298 to 80 K. For PL measurements: excitation at 806 and 781 nm for Au_42_ and Au_42_/DMBA (857.6 mM), respectively, slit width 8 nm, and emission slit 8 nm. (C) Variation of QY of FL for Au_42_ and Au_42_/DMBA from 298 to 80 K. (D) Plot of *k*_*r*_ (green symbols) and *k*_*nr*_ (yellow symbols) from 80 to 298 K. (E) Normalized integrated intensities of FL for Au_42_ and Au_42_/DMBA and fitting using eq 1 (data from panel C). (F) fwhm of the FL as a function of temperature for Au_42_ and Au_42_/DMBA and fitting using eq 2. Both eqs 1 and 2 are in the text.

We further compared the *k*_*r*_ and *k*_*nr*_ of the FL for both Au_42_ and Au_42_/DMBA systems at low temperatures (Figure 2D). The *k*_*r*_ values for both systems remain relatively constant, but the *k*_*nr*_ values for both Au_42_ and Au_42_/DMBA exhibit a notable decrease, attributed to the suppression of staple vibrations at low temperatures; note: the core vibrations are typically manifested at even lower temperatures than 80 K.^49^ Additionally, it is important to highlight that the *k*_*nr*_ of Au_42_/DMBA is significantly lower than that of Au_42_ at the same temperatures. To elucidate the mechanism underlying the decrease in *k*_*nr*_ of FL upon the addition of DMBA, we fitted the temperature-dependent FL intensity evolution by eq 1(50)
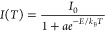
1where *I*_0_ represents the initial intensity, *a* denotes the ratio of nonradiative and radiative probabilities, and *E* is the activation energy for the nonradiative relaxation. Here, only one dominant phonon-assisted nonradiative channel is considered in this modeling. The corresponding fitting line and parameters are shown in Figure 2E, where the activation energies of phonon modes that coupled with the FL of Au_42_ and Au_42_/DMBA are determined to be 38.9 and 22.3 meV, respectively; note: 1 meV = 8 cm^–1^. This suggests that the addition of DMBA suppresses the vibrations associated with the Au–S staples on the Au_42_. Meanwhile, the *a* value falls drastically from 4.5 to 1.6, also indicating a significant suppression of the staple vibration-induced nonradiative decay. Moreover, we extracted and compared the temperature-dependent full-width at half-maximum (fwhm) values for Au_42_ and Au_42_/DMBA (Figure 2F). Generally, both acoustic phonon modes (low energy) and optical phonon modes (high energy) contribute to the broadening of PL line width, but our experiments are conducted down to 80 K only — where the contributions from acoustic phonons are trivial and can be omitted, thus we only consider the optical phonon factor to model the line width broadening by eq 2(24)
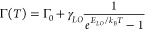
2where Γ_0_ is the temperature-independent intrinsic line width, *γ*_*LO*_ refers to the coupling coefficient of electrons with longitudinal optical (LO) phonons, and *E*_*LO*_ denotes the average energy for coupled LO phonon modes. The modeling results (Figure 2F) reveal that the average LO phonon energies for Au_42_ and Au_42_/DMBA are 30 and 15 meV, respectively. The reduced phonon energy in Au_42_/DMBA aligns with the eq 1 fitting analysis, indicating a suppression of surface vibrations. Meanwhile, the coupling strength for Au_42_/DMBA (*γ*_*LO*_ = 43 meV) is much lower than that for Au_42_ (*γ*_*LO*_ = 132 meV), suggesting a diminished electron–phonon interaction in the Au_42_/DMBA system.

The high PLQY (50%) of Au_42_/DMBA in the NIR region is rare among the reported Au NCs (Figure S10). In addition to DMBA, we found that other amide molecules (Figure 3A), such as *N,N*-dimethylformamide (DMF), *N,N*-dimethylacetamide (DMAc), and *N*-methylformanilide (NMFA), have similar effects on Au_42_, including (i) the longitudinal absorption peak of Au_42_ at 806 nm undergoes a blueshift when mixed with these molecules (Figure S11), and (ii) a significant enhancement of the PLQY of Au_42_ is observed (Figure S12 and Table S4), e.g., 29.3% for DMF, 47.0% for DMAc, and 55.8% for NMFA. The peak deconvolution analysis (Figure S13) further indicates that these amides predominantly boost the FL (Figure 3B) but not the PH. Additionally, the observed increase in FL intensity is primarily attributed to the suppression of nonradiative relaxation (Figure 3B).

**Figure 3:**
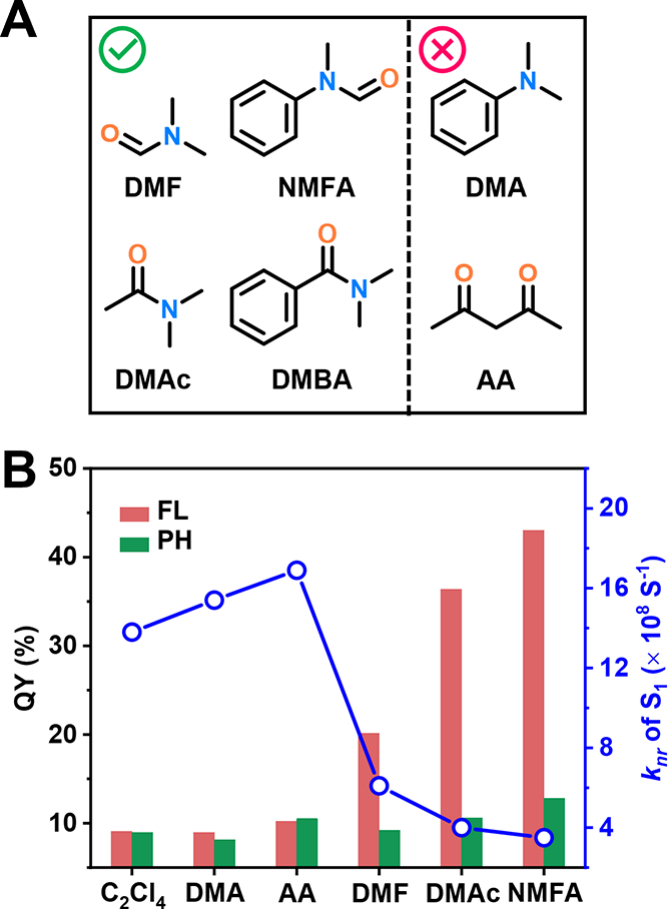
(A) Structures of different small molecules. (B) QYs (bars) and *k*_*nr*_ (blue symbols) for Au_42_ mixed with different molecules.

To pinpoint the specific atoms in the amide group accountable for the PL enhancement, we tested two small molecules composed of only nitrogen or oxygen atom, e.g., *N,N*-dimethylaniline (DMA) and acetylacetone (AA), but neither molecule nor their mixture induced any blueshift in the longitudinal absorption peak of Au_42_ (Figure S14), nor did they enhance the PL intensity of Au_42_ (Figure S15 and Table S4). This comparison underscores a cooperative effect of nitrogen and oxygen atoms of amides on the PL enhancement of Au_42_ while retaining its structure (Figure S16).

The amide molecules can further enhance the emission of Au_42_ embedded in a polymer film. As illustrated in Figure S17, the PLQY of sole Au_42_ increases from 18% to 52% when embedded in polystyrene (PS) films, and it is further elevated to 75% with the addition of DMBA into the Au_42_/PS film at room temperature. This highly emissive film holds promise in applications such as NIR optoelectronic devices and security as well as quantum telecom.

In summary, we report an effective strategy involving noncoordinative interactions between amides and Au_42_ to achieve high PLQY (50% in solutions and 75% in films) in the NIR range by significantly reducing the nonradiative decay rate. This method is also effective for other Au_*n*_ quantum rods.^48^ Our findings offer inspirations for strategically designing highly efficient NIR emitters, opening new avenues for the use of engineered nanoclusters in diverse applications.

## Supporting Information Available

Details of the synthesis and data analysis and additioanl figures and tables as described in the text (PDF)
