# Supplementary material for: Raising Near-Infrared Photoluminescence Quantum Yield of Au42 Quantum Rod to 50% in Solutions and 75% in Films
Source: J Am Chem Soc. 2024 Oct 3;146(41):27993–7. doi: 10.1021/jacs.4c11703 (PMC11487566; doi:10.1021/jacs.4c11703)
Supplement: Supplementary file 1 — ja4c11703_si_001.pdf [file ja4c11703_si_001.pdf]

Supporting Information:

Raising Near-Infrared Photoluminescence Quantum Yield of Au_{42} Quantum Rod to 50% in Solutions and 75% in Films

Lianshun Luo,[†,#] Zhongyu Liu,[†,#] Abhrojyoti Mazumder,[†] and Rongchao Jin[†,*]

[†]Department of Chemistry, Carnegie Mellon University, Pittsburgh PA 15213, United States

*To whom correspondence should be addressed: rongchao@andrew.cmu.edu (R.J.)

[#]L.L. and Z.L. contributed equally to this work.

Chemicals

Tetrachloroauric (III) acid ($HAuCl_4 \cdot 3H_2O$, 99.999% metal basis, Aldrich), phenylethyl mercaptan (PET, >99%, Aldrich), sodium borohydride (powder, ≥98%, Sigma), benzimidazole (98%, Aldrich), 2-bromopropane (99%, Aldrich), dimethyl sulfide (SMe_2, >99%, Aldrich), potassium carbonate (K_2CO_3, 99%, Aldrich), *N*,*N*-dimethylbenzamide (DMBA, 99%, Sigma), 2-methyltetrahydrofuran (2-MeTHF), tetrachloroethylene (C_2Cl_4), *N*,*N*-dimethylformamide (DMF), *N*,*N*-dimethylacetamide (DMAc), *N*-methylformanilide (NMFA), *N*,*N*-dimethylaniline (DMA) and acetylacetone (AA), methanol, ethanol, ethyl ether, ethyl acetate, pentane, dichloromethane (DCM), chloroform, toluene, acetone, *n*-hexane and acetonitrile (HPLC grade for all solvents) were used as received. Thin-layer chromatography (TLC) plates were purchased from iChromatography (silica gel, 250 μm).

Synthesis of chloro(dimethylsulfide)gold(I) ($AuCl(SMe_2)$)

$HAuCl_4 \cdot 3H_2O$ (500 mg, 1.27 mmol) was dissolved in ethanol (20 mL), followed by the addition of SMe_2 (280 μL, 3.81 mmol), and the solution was vigorously stirred for 2 hours. After that, the white precipitate was collected by centrifugation, which was washed with ethyl ether and finally dried to give the product as a white powder.

Synthesis of 1,3-diisopropylbenzimidazolium bromide (iPr$_2$-bimy·HBr)

Benzimidazole (1.18 g, 10 mmol) and K_2CO_3 (760 mg, 5.5 mmol) were added into acetonitrile (8 mL) and the mixture was rapidly stirred at ambient temperature for 1 hour. Following that, 2-bromopropane (5.4 mL, 57.5 mmol) was added to the suspension, and the reaction mixture was vigorously stirred under reflux conditions for 24 hours, followed by the addition of a second portion of 2-bromopropane (5.4 mL, 57.5 mmol). The reaction mixture was vigorously stirred under reflux for additional 48 hours. After removing the solvent under reduced pressure, DCM was added to the residues, and the upper supernatant after centrifugation was collected. The solvent of the supernatant was removed under reduced pressure to produce a spongy solid, which was washed by ethyl acetate to afford the desired product as a white powder.

Synthesis of NHC-Au-Br complex ($^{i}Pr_2$-bimy·AuBr)

$^{i}Pr_2$-bimy·HBr (1337.4 mg, 4.725 mmol), $AuCl(SMe_2)$ (1393.4 mg, 4.725 mmol), and K_2CO_3 (653.5 mg, 4.725 mmol) were added into acetone (20 mL) and the mixture was vigorously stirred under reflux

conditions for 2 hours. After that, the solvent in the suspension was removed under reduced pressure. DCM was added to the residues, and the upper supernatant after centrifugation was collected. The solvent of the supernatant was removed under reduced pressure to give the solid product, which was washed with pentane and finally dried to afford the desired product as a gray powder.

Synthesis of $Au_{42}(PET)_{32}$

$^{i}Pr_2$-bimy·AuBr (120 mg, 0.25 mmol) and PET (67 μL, 0.5 mmol) were dissolved in a mixture of chloroform (15 mL) and ethanol (5 mL). The mixture gradually turned cloudy white (Au^{I}-PET formed). After 30 min of stirring, the suspension was reduced to nanoclusters (NCs) by the addition of sodium borohydride (95 mg, 2.5 mmol, dissolved in 5 mL ice-cold H_2O) under darkness. A black solution was formed. The reaction was continued for 5 hours, and then the solvent was removed under reduced pressure. The mixture of Au NCs was thoroughly washed with methanol, extracted with DCM, and concentrated for TLC separation. The mixture of Au NCs was pipetted onto a TLC plate, and separation was conducted in a developing tank (developing solvent 1:1 (v/v) DCM:*n*-hexane). The green band corresponding to $Au_{42}(PET)_{32}$ was cut off and dissolved in DCM for characterization.

Preparation of Au_{42}/polystyrene thin films

The drop-cast method was used to prepare the solid thin films. First, a stock solution of Au_{42} was prepared by dissolving Au_{42} (1 mg) in toluene (1 mL). Meanwhile, a polystyrene stock solution was prepared by dissolving polystyrene (PS, 10 mg) in toluene (1 mL). Then, 20 μL of Au_{42} solution and 38 μL of polystyrene solution were mixed to afford an ink-like solution. Finally, the solution was dropped on a quartz plate and dried slowly at room temperature to give a Au_{42}/PS film in which the mass fraction of Au_{42} is 5 wt%.

Preparation of (Au_{42}/DMBA)/polystyrene thin films

The drop-cast method was used to prepare the solid thin films. First, a stock solution of Au_{42} was prepared by dissolving Au_{42} (1 mg) in 2-MeTHF containing DMBA (857.6 mM, 1 mL). Meanwhile, a polystyrene stock solution was prepared by dissolving PS (10 mg) in 2-MeTHF (1 mL). Then, 20 μL of Au_{42}/DMBA solution and 38 μL of polystyrene solution were mixed to afford an ink-like solution. Finally, the solution was dropped on a quartz plate and dried slowly at room temperature to offer a (Au_{42}/DMBA)/PS film in which the mass fraction of Au_{42} is 5 wt%.

Steady-state UV-Vis-NIR and cryogenic measurements

UV-Vis-NIR spectra of Au NCs were collected on a UV-3600 Plus UV-VIS-NIR spectrophotometer (Shimadzu). The cryogenic spectra (room temperature to 80 K) were recorded on a home-built system including the UV-3600 spectrometer, a vacuum pump, an Optistat CF2 cryostat (Oxford Instruments) and a temperature controller. Liquid nitrogen was used as the cryogen.

Steady-state and time-resolved photoluminescence and cryogenic measurements

Steady state photoluminescence spectra were measured on a FLS-1000 spectrofluorometer (Edinburgh). Near infrared PL was measured using a wide-range photomultiplier tube (PMT-1700, up to ~1700 nm

wavelength) cooled to -80 ℃ by liquid nitrogen.

The PL lifetimes were measured by time-correlated single photon counting (TCSPC) on the same instrument.

Cryogenic PL and PLE spectra were measured on a home-built system, which includes the FLS-1000 spectrofluorometer, a vacuum pump, an Optistat CF2 cryostat (Oxford Instruments) and a temperature controller. Liquid nitrogen was used as the cryogen.

The QYs of Au_{42} in solutions at room temperature were measured by an integrating sphere. The QYs of Au_{42} films at room temperature were determined by the comparison of emission intensities of Au_{42} films and Au_{42} solutions. The QYs at cryogenic temperatures were determined by the comparison of emission intensities at room temperature and lower temperatures with the low-temperature enhanced absorption corrected.

Supporting figures:

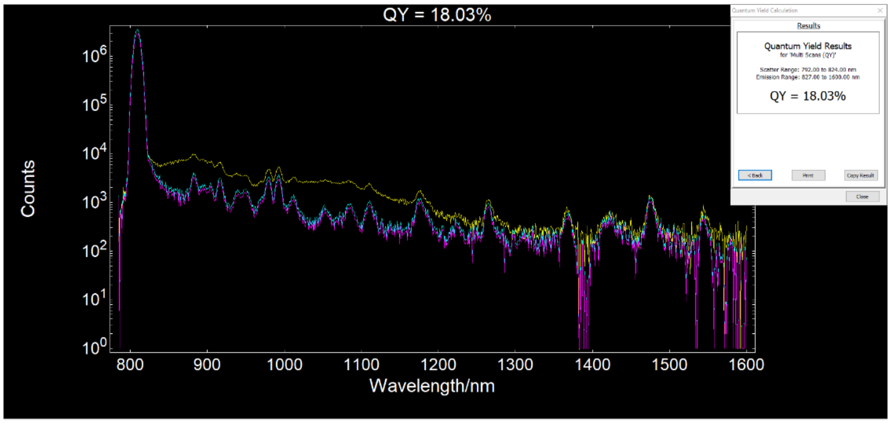

Figure S1. QY measurement of Au_{42} dissolved in C_2Cl_4 (excitation: 806 nm, slit width 12 nm, and emission slit 6.7 nm).

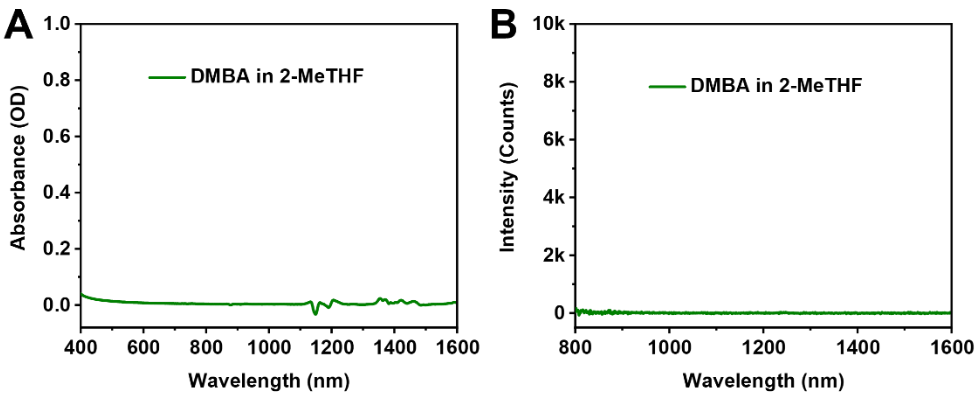

Figure S2. (A) Optical absorption and (B) PL spectra of DMBA dissolved in 2-MeTHF. For PL measurements, excitation at 400 nm, slit width 8 nm, and emission slit 8 nm.

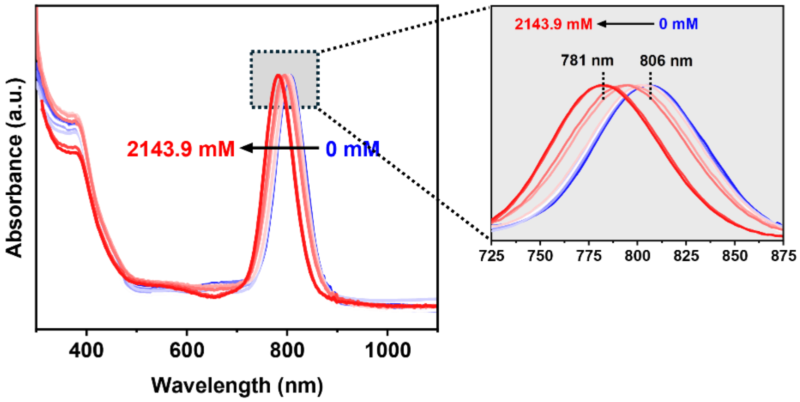

Figure S3. Optical absorption spectra of Au_{42} dissolved in deaerated 2-MeTHF containing DMBA with different concentrations (0 to 2143.9 mM).

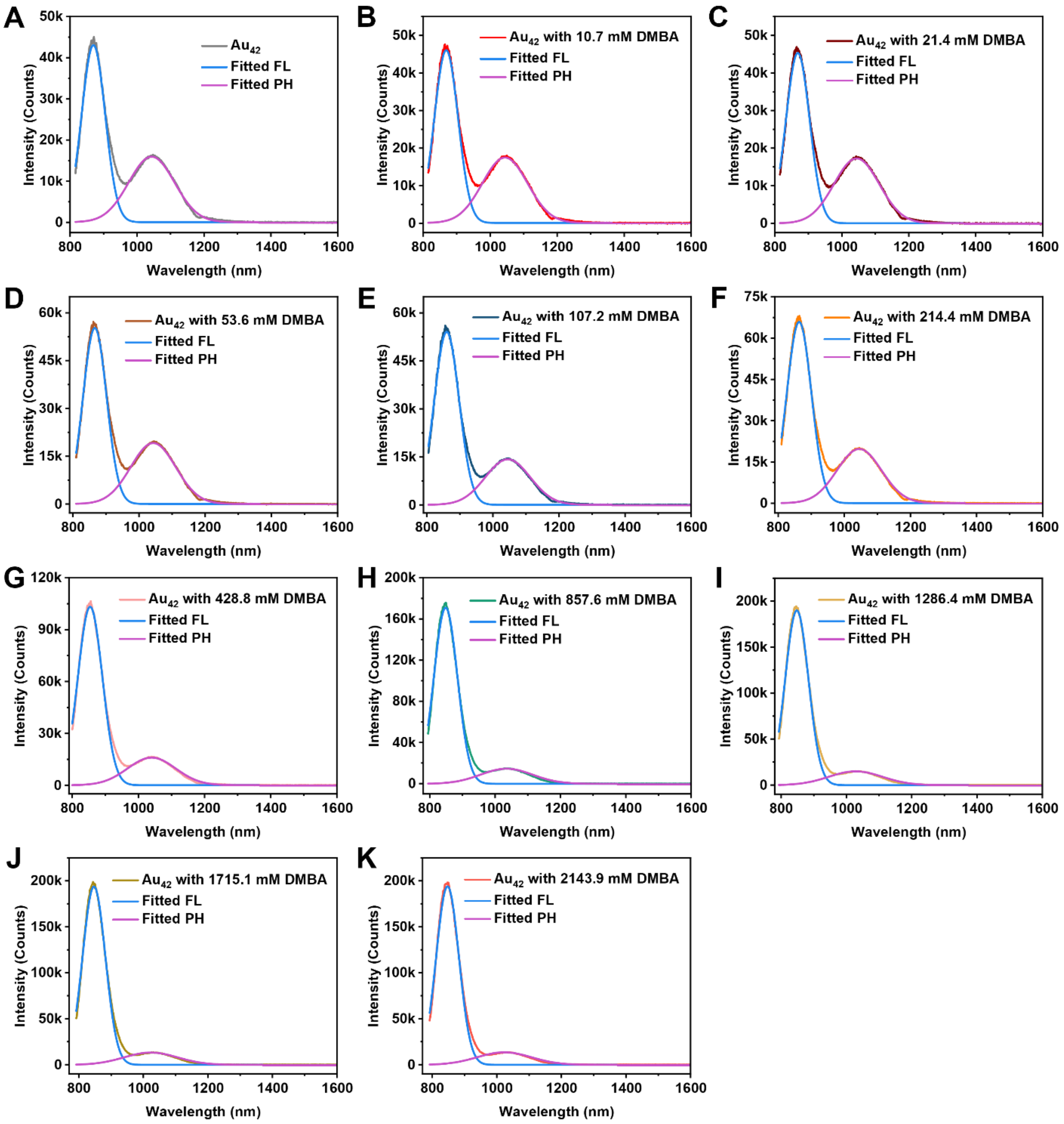

Figure S4. PL spectra and peak deconvolution for Au_{42} dissolved in deaerated 2-MeTHF containing DMBA with different concentrations. Blue and Purple curves in all figures correspond to the fitted FL and PH, respectively.

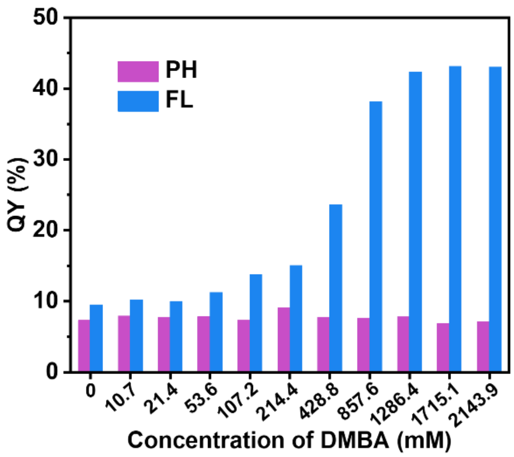

Figure S5. QYs of FL and PH for Au_{42} dissolved in deaerated 2-MeTHF containing DMBA with different concentrations.

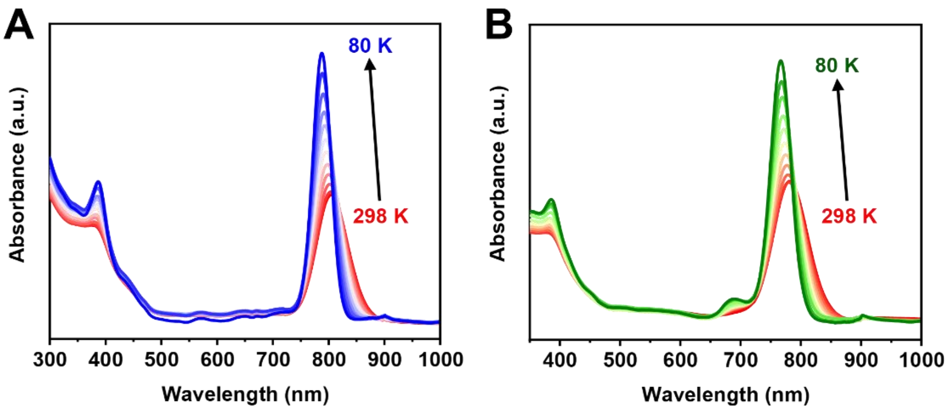

Figure S6. Temperature-dependent absorption profiles of Au_{42} (A) without DMBA, and (B) with DMBA (857.6 mM) dissolved in deaerated 2-MeTHF.

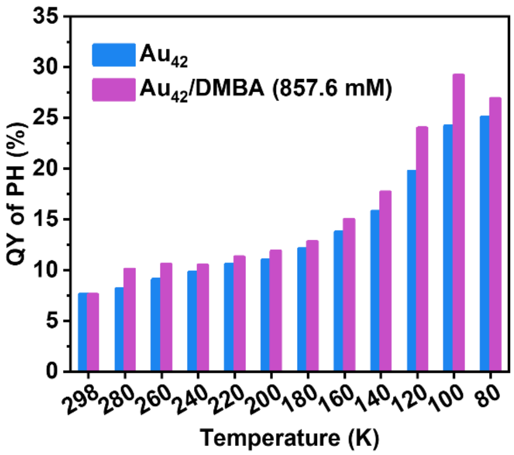

Figure S7. QYs of PH for Au_{42} with and without DMBA (857.6 mM) dissolved in deaerated 2-MeTHF at different temperatures.

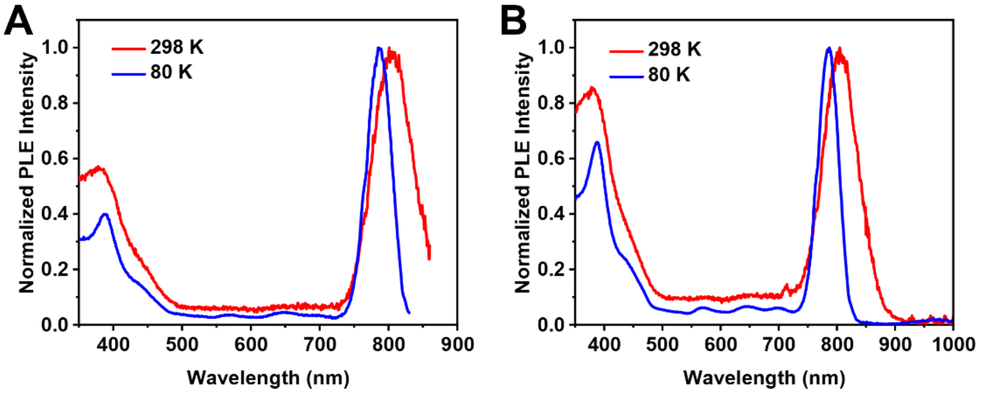

Figure S8. Excitation spectra for FL (A) and PH (B) of Au_{42} at room temperature and 80 K (red and blue curves).

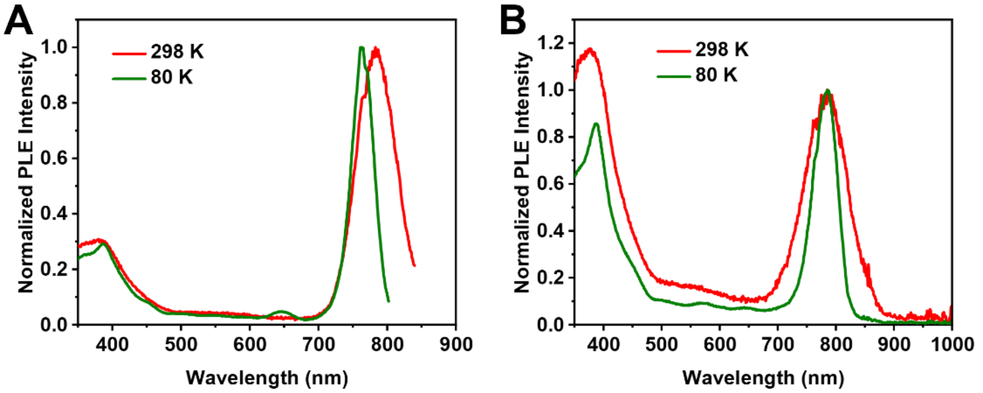

Figure S9. Excitation spectra for FL (A) and PH (B) of Au_{42} with DMBA (857.6 mM) at room temperature and 80 K (red and green curves).

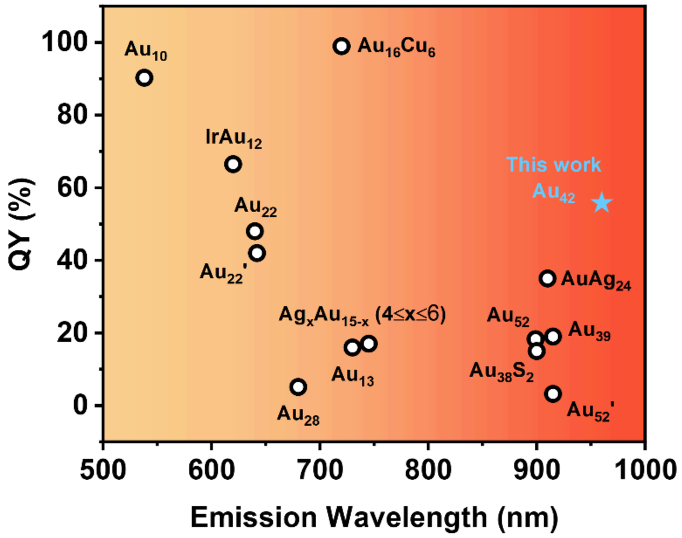

Figure S10. Comparison of PL properties of Au_{42} in this work with other highly emissive nanoclusters reported in refs 1-13 (listed in the end of SI).

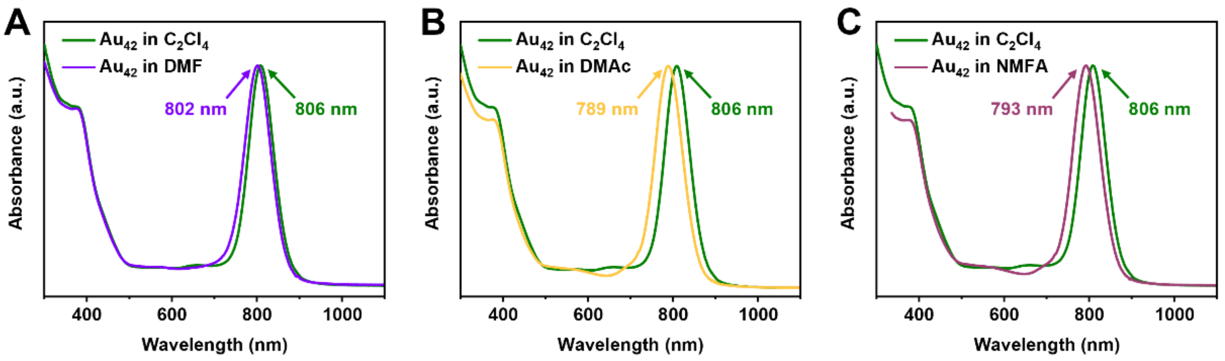

Figure S11. Optical absorption spectra for Au_{42} dissolved in (A) DMF, (B) DMAc, and (C) NMFA. All show a small blueshift of the original 806 nm peak.

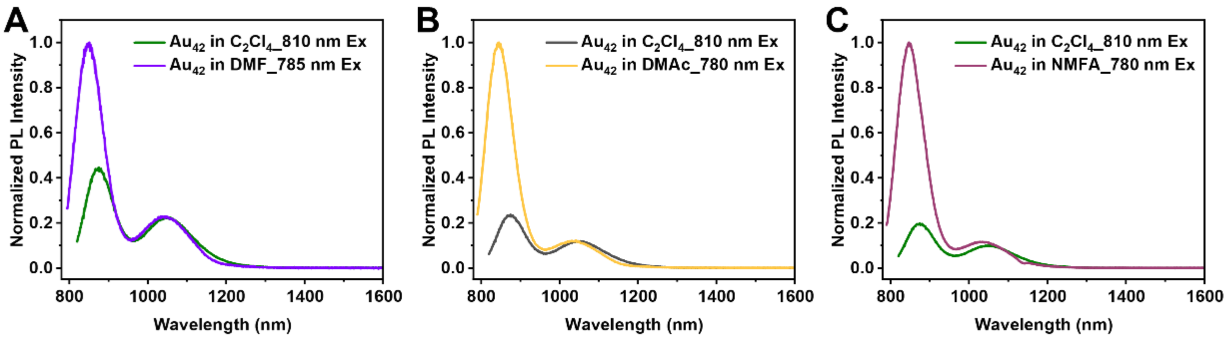

Figure S12. PL spectra for Au_{42} dissolved in (A) DMF, (B) DMAc, and (C) NMFA. For PL measurements: under a N_2 atmosphere, excitation slit width 8 nm, and emission slit 8 nm.

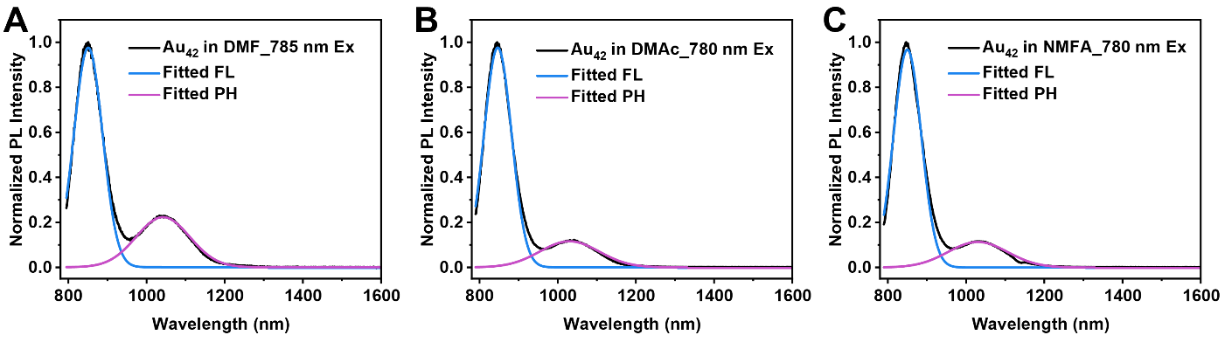

Figure S13. PL spectra (black) and peak deconvolution (blue and purple curves for the fitted FL and PH, respectively) for Au_{42} dissolved in (A) DMF, (B) DMAc, and (C) NMFA.

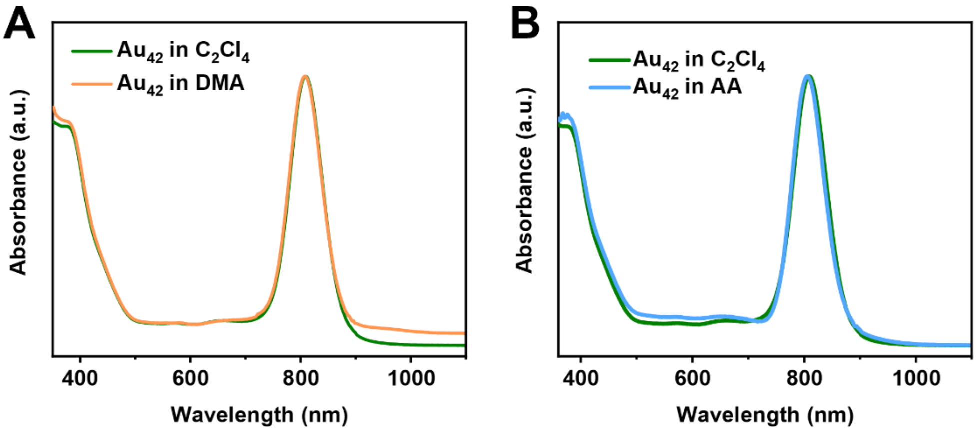

Figure S14. Optical absorption spectra of Au_{42} dissolved in (A) DMA and (B) AA. Similarly, no spectral change was seen with a mixture of DMA and AA (1:1 ratio) added to Au_{42}.

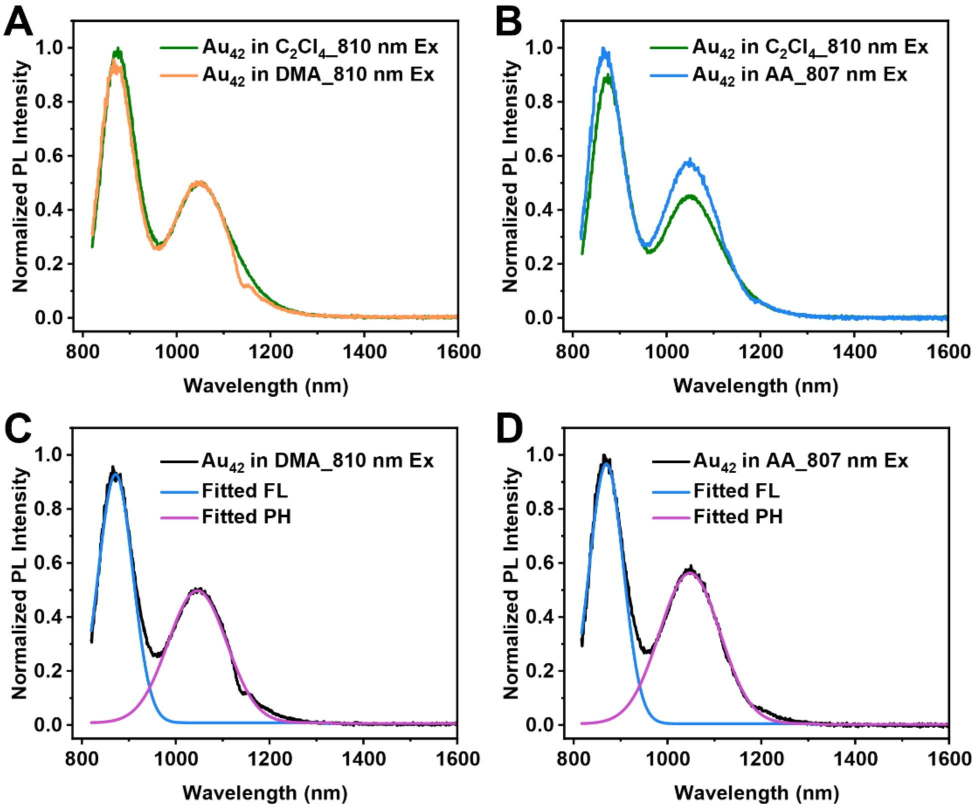

Figure S15. PL spectra for Au_{42} dissolved in (A) DMA and (B) AA (both in comparison with Au_{42} in C_2Cl_4). Peak deconvolution for Au_{42} in (C) DMA and (D) AA (where, blue and purple curves correspond to the fitted FL and PH, respectively). Similarly, no PL enhancement was observed with a mixture of DMA and AA (1:1 ratio) added to Au_{42}. For PL measurements: under a N_2 atmosphere, excitation slit width 8 nm, and emission slit 8 nm.

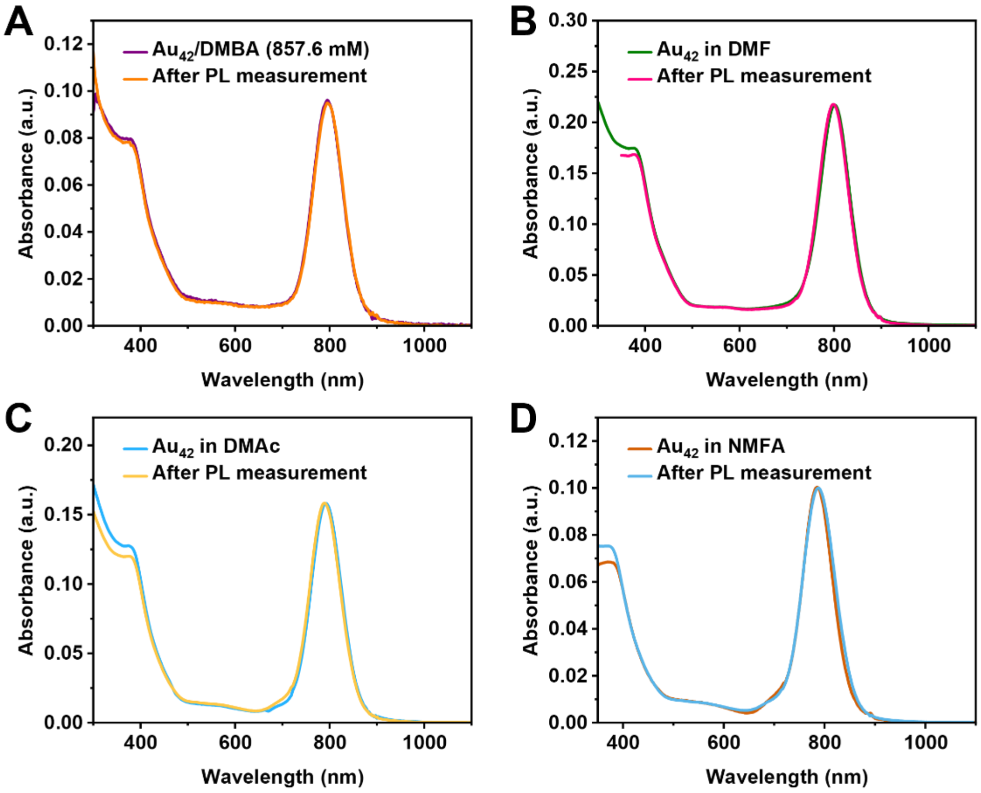

Figure S16. Optical absorption spectra for Au_{42} dissolved in (A) DMBA (857.6 mM)/2-MeTHF, (B) DMF, (C) DMAc, and (D) NMFA before and after PL measurements. No degradation was seen.

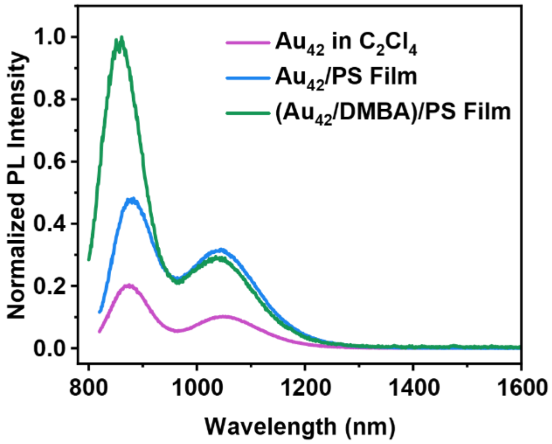

Figure S17. PL spectra of Au_{42} dissolved in C_2Cl_4 and embedded in PS film. For PL measurements: under a N_2 atmosphere, excitation slit width 8 nm, and emission slit 8 nm, excitation at 806 nm for Au_{42} dissolved in C_2Cl_4 and embedded in PS film without DMBA, excitation at 790 nm for Au_{42} embedded in PS film with DMBA.

Table S1. Parameters of the PL properties for Au_{42} mixed with DMBA at different concentrations in deaerated 2-MeTHF. Note: k_r and k_{nr} were calculated by $k_r = \Phi_{FL}\cdot\tau^{-1}$, $k_{nr} = (1 - \Phi_{FL})\cdot\tau^{-1}$.

DMBA Conc. (mM)	Normalized PL Intensity*	Total QY (%)	QY of FL (%)	QY of PH (%)	τ of S_1 (ns)	k_r of S_1 (× 10^8 s^{-1})	k_{nr} of S_1 (× 10^8 s^{-1})
0	1.00 fixed	16.8	9.5	7.3	0.67	1.42	13.51
10.7	1.08	18.1	10.2	7.9	0.64	1.59	14.03
21.4	1.05	17.7	10.0	7.7	0.67	1.49	13.43
53.6	1.14	19.1	11.2	7.9	0.65	1.73	13.66
107.2	1.26	21.1	13.8	7.3	0.70	1.97	12.32
214.4	1.43	24.1	15.0	9.1	0.73	2.06	11.64
428.8	1.87	31.4	23.6	7.8	0.85	2.78	8.98
857.6	2.72	45.7	38.1	7.6	1.28	2.98	4.83
1286.4	2.99	50.2	42.4	7.8	1.50	2.82	3.84
1715.1	2.98	50.0	43.1	6.9	1.69	2.55	3.37
2143.9	2.98	50.1	43.0	7.1	1.71	2.51	3.33

* The values are taken from the cryogenic absorption measurements (see Fig. 1D in main text)

Table S2. Parameters of the cryogenic PL properties of Au_{42} in deaerated 2-MeTHF.

Temperature (K)	Normalized PL Intensity	Total QY (%)	QY of FL (%)	QY of PH (%)	τ of S_1 (ns)	k_r of S_1 (× 10^8 s^{-1})	k_{nr} of S_1 (× 10^8 s^{-1})
298	1.00 fixed	16.8	9.5	7.3	0.67	1.42	13.51
280	1.11	18.6	10.5	8.1	–	–	–
260	1.24	20.8	11.7	9.1	0.85	1.38	10.38
240	1.34	22.6	12.8	9.8	–	–	–
220	1.44	24.3	13.8	10.5	1.03	1.33	8.36
200	1.50	25.3	14.3	11.0	–	–	–
180	1.63	27.5	15.4	12.1	1.16	1.33	7.32
160	1.78	29.9	16.2	13.7	–	–	–
140	1.95	32.7	16.9	15.8	1.21	1.40	6.88
120	2.26	38.0	18.2	19.8	–	–	–
100	2.58	43.3	19.1	24.2	1.21	1.57	6.67
80	2.71	45.6	20.5	25.1	1.23	1.67	6.44

Table S3. Parameters of the cryogenic PL properties of Au_{42} mixed with DMBA (857.6 mM) in deaerated 2-MeTHF.

Temperature (K)	Normalized PL Intensity	Total QY (%)	QY of FL (%)	QY of PH (%)	τ of S_1 (ns)	k_r of S_1 ($\times 10^8$ s^{-1})	k_{nr} of S_1 ($\times 10^8$ s^{-1})
298	1.00 fixed	45.7	38.1	7.6	1.28	2.98	4.83
280	1.02	46.5	36.4	10.1	–	–	–
260	1.11	50.7	40.0	10.6	2.09	1.92	2.87
240	1.13	51.7	41.2	10.5	–	–	–
220	1.19	54.5	43.2	11.3	2.29	1.88	2.48
200	1.22	55.9	44.0	11.9	–	–	–
180	1.25	57.2	44.4	12.8	2.46	1.81	2.26
160	1.36	62.1	47.1	15.0	–	–	–
140	1.44	65.9	48.2	17.7	2.48	1.95	2.09
120	1.65	75.3	51.3	24.0	–	–	–
100	1.85	84.5	55.3	29.2	2.49	2.22	1.80
80	1.95	89.1	62.2	26.9	2.48	2.51	1.52

Table S4. Parameters of the PL properties of Au_{42} dissolved in different solvents.

Solvents	Normalized PL Intensity	Total QY (%)	QY of FL (%)	QY of PH (%)	τ of S_1 (ns)	k_r of S_1 ($\times 10^8$ s^{-1})	k_{nr} of S_1 ($\times 10^8$ s^{-1})
C_2Cl_4	1.00 fixed	18.0	9.1	8.9	0.66	1.37	13.8
DMA	0.95	17.1	8.9	8.2	0.59	1.52	15.4
AA	1.15	20.7	10.2	10.5	0.53	1.92	16.9
DMF	1.63	29.3	20.1	9.2	1.32	1.52	6.1
DMAc	2.61	47.0	36.4	10.6	1.59	2.29	4.0
NMFA	3.10	55.8	43.0	12.8	1.63	2.64	3.5

References:

1. Zhong, Y.; Zhang, J.; Li, T.; Xu, W.; Yao, Q.; Lu, M.; Bai, X.; Wu, Z.; Xie, J.; Zhang, Y., Suppression of kernel vibrations by layer-by-layer ligand engineering boosts photoluminescence efficiency of gold nanoclusters. *Nat. Commun.* **2023,** *14*, 658.

2. Pyo, K.; Ly, N. H.; Yoon, S. Y.; Shen, Y.; Choi, S. Y.; Lee, S. Y.; Joo, S. W.; Lee, D., Highly Luminescent Folate-Functionalized Au_{22} Nanoclusters for Bioimaging. *Adv. Healthcare Mater.* **2017,** *6*, 1700203.

3. Deng, H.; Huang, K.; Xiu, L.; Sun, W.; Yao, Q.; Fang, X.; Huang, X.; Noreldeen, H. A. A.; Peng, H.; Xie, J.; Chen, W., Bis-Schiff base linkage-triggered highly bright luminescence of gold nanoclusters in aqueous solution at the single-cluster level. *Nat. Commun.* **2022,** *13*, 3381.

4. Zhu, C.; Chen, Z. L.; Li, H.; Lu, L.; Kang, X.; Xuan, J.; Zhu, M., Rational Design of Highly Phosphorescent Nanoclusters for Efficient Photocatalytic Oxidation. *J. Am. Chem. Soc.* **2024**, doi: /10.1021/jacs.4c05530.

5. Wang, Y.; Liu, Z.; Mazumder, A.; Gianopoulos, C. G.; Kirschbaum, K.; Peteanu, L. A.; Jin, R., Tailoring Carbon Tails of Ligands on $Au_{52}(SR)_{32}$ Nanoclusters Enhances the Near-Infrared Photoluminescence Quantum Yield from 3.8 to 18.3%. *J. Am. Chem. Soc.* **2023,** *145*, 26328-26338.

6. Liu, Z.; Luo, L.; Kong, J.; Kahng, E.; Zhou, M.; Jin, R., Bright near-infrared emission from the $Au_{39}(SR)_{29}$ nanocluster. *Nanoscale* **2024,** *16*, 7419-7426.

7. Pniakowska, A.; Kumaranchira Ramankutty, K.; Obstarczyk, P.; Peric Bakulic, M.; Sanader Marsic, Z.; Bonacic-Koutecky, V.; Burgi, T.; Olesiak-Banska, J., Gold-Doping Effect on Two-Photon Absorption and Luminescence of Atomically Precise Silver Ligated Nanoclusters. *Angew. Chem. Int. Ed.* **2022,** *61*, e202209645.

8. Shi, W.-Q.; Zeng, L.; He, R.-L.; Han, X.-S.; Guan, Z.-J.; Zhou, M.; Wang, Q.-M. Near-Unity NIR Phosphorescent Quantum Yield from a Room-Temperature Solvated Metal Nanocluster. *Science* **2024**, *383*, 326-330.

9. Li, Q.; Zeman, C. J. t.; Schatz, G. C.; Gu, X. W., Source of Bright Near-Infrared Luminescence in Gold Nanoclusters. *ACS Nano* **2021,** *15*, 16095-16105.

10. Narouz, M. R.; Takano, S.; Lummis, P. A.; Levchenko, T. I.; Nazemi, A.; Kaappa, S.; Malola, S.; Yousefalizadeh, G.; Calhoun, L. A.; Stamplecoskie, K. G.; Hakkinen, H.; Tsukuda, T.; Crudden, C. M., Robust, Highly Luminescent Au_{13} Superatoms Protected by N-Heterocyclic Carbenes. *J. Am. Chem. Soc.* **2019,** *141*, 14997-15002.

11. Hirai, H.; Takano, S.; Nakashima, T.; Iwasa, T.; Taketsugu, T.; Tsukuda, T., Doping-Mediated Energy-Level Engineering of M@Au_{12} Superatoms (M=Pd, Pt, Rh, Ir) for Efficient Photoluminescence and Photocatalysis. *Angew. Chem. Int. Ed.* **2022,** *61*, e202207290.

12. Gu, W.; Zhou, Y.; Wang, W.; You, Q.; Fan, W.; Zhao, Y.; Bian, G.; Wang, R.; Fang, L.; Yan, N.; Xia, N.; Liao, L.; Wu, Z., Concomitant Near-Infrared Photothermy and Photoluminescence of Rod-Shaped $Au_{52}(PET)_{32}$ and $Au_{66}(PET)_{38}$ Synthesized Concurrently. *Angew. Chem. Int. Ed.* **2024,** *63*, e202407518.

13. Dong, J.; Gan, Z.; Gu, W.; You, Q.; Zhao, Y.; Zha, J.; Li, J.; Deng, H.; Yan, N.; Wu, Z., Synthesizing Photoluminescent $Au_{28}(SCH_2Ph\text{-}^tBu)_{22}$ Nanoclusters with Structural Features by Using a Combined Method. *Angew. Chem. Int. Ed.* **2021,** *60*, 17932-17936.
